# Investigation of Temperature-Field Evolution and Microstructural Response in Bituminous Waterproofing Membranes Under Low-Temperature Flexibility Testing Conditions

**DOI:** 10.3390/polym18111294

**Published:** 2026-05-25

**Authors:** Jun Tan, Lei Geng, Dong Zhang, Chen Li, Chao Zhang

**Affiliations:** 1 China Electronics Engineering Design Institute Co., Ltd., Beijing 100142, China; tanjun@ceedi.cn (J.T.); zhangchaotim@163.com (C.Z.); 2 China National Accreditation Institute of Conformity Assessment, Beijing 100062, China; gengl@cnas.org.cn; 3 China Institute of Building Standard Design and Research, Beijing 100142, China; 248zd@163.com

**Keywords:** waterproofing membrane, low-temperature conditioning, temperature field, heat transfer lag effect, finite element analysis (FEA)

## Abstract

Low-temperature conditioning is a key procedure in the flexibility evaluation of waterproofing membranes and directly affects the reliability of subsequent performance assessments. However, the internal unsteady-state heat transfer kinetics and the thermal gradient evolution mechanisms in multi-layer composite membranes under transient cold shocks require further investigation. Focusing on commonly utilized 3 mm and 4 mm thick SBS (Styrene–Butadiene–Styrene)-modified bitumen waterproofing membranes as subjects, this study investigated the internal dynamic temperature fields and microstructural response of bituminous waterproofing membranes under standard low-temperature flexibility testing conditions. By accurately pre-embedding micro-temperature sensors in situ at the interface between the surface layer and the reinforcement matrix, the transient thermal response profiles of specimens with varying specifications in a −25 °C liquid environment were quantified. Simultaneously, a three-dimensional transient heat conduction finite element model was established to elucidate the dynamic evolution of internal spatial temperature gradients. The congruence between experimental and numerical results demonstrates that upon exposure to extreme cold, composite membranes of different thicknesses exhibit a pronounced “surface-to-core” heat transfer lag effect. The cooling rate maximized within the initial 10 min of exposure. Conversely, the internal interface layer—acting as a high-thermal-resistance zone and the most unfavorable point for heat conduction—necessitated 10~20 min of nonlinear thermal dissipation to stabilize at the target ambient temperature. This study clarifies the transient thermal response and temperature-field evolution laws of bituminous waterproofing membranes, providing a robust theoretical framework for elucidating low-temperature embrittlement mechanisms and informing the material design and application of waterproofing projects in cold regions.

## 1. Introduction

With the continuous expansion of infrastructure development in cold regions, the low-temperature durability of waterproofing systems is confronted with severe challenges. As a fundamental barrier safeguarding the structural integrity of buildings, bituminous waterproofing membranes are highly prone to glass transition within their polymer matrix under extreme low-temperature alternating environments. This phenomenon triggers a loss of strain compatibility, which subsequently facilitates microcrack initiation or even induces brittle fracture, ultimately precipitating waterproofing system failure and compromising the structure through leakage [1]. Therefore, an in-depth investigation and rigorous assessment of low-temperature flexibility evolution are of critical practical significance for extending the service life of infrastructure in cold regions.

Currently, the evaluation of low-temperature performance for waterproofing membranes predominantly relies on macroscopic mechanical tests premised on the assumption of “quasi-steady-state thermal equilibrium,” such as laboratory low-temperature bending tests [2]. However, bituminous waterproofing membranes utilized in engineering typically feature multi-layer composite heterogeneous architectures, consisting of asphalt coating layers and reinforcement matrices such as polyester or glass fiber. Significant discrepancies exist in the thermophysical properties, such as thermal conductivity and specific heat capacity, across these constituent layers [3]. Upon exposure to cryogenic environments and transient cold shocks, complex non-steady-state thermal gradients inevitably evolve within the material matrix. The existing literature often overlooks this transient heat transfer process, resulting in a paucity of quantitative analysis regarding the spatio-temporal evolution of internal temperature and the heat transfer lag effect in multi-layer composite structures [4,5]. This opaque heat transfer mechanism not only impedes a fundamental understanding of microscopic embrittlement and mechanical attenuation mechanisms driven by thermal gradients but also precludes the establishment of reliable thermodynamic boundary conditions for evaluating the real service state of materials under complex operating conditions in cold regions.

In recent years, extensive research has been conducted worldwide on the low-temperature degradation behavior of modified asphalt and waterproofing membranes. Existing studies have mainly focused on two aspects: the microstructural mechanisms of material aging and the macroscopic evaluation of mechanical performance. At the microscale, techniques such as dynamic mechanical analysis (DMA), Fourier-transform infrared spectroscopy (FTIR), and scanning electron microscopy (SEM) have been widely employed to characterize the evolution of functional groups, phase degradation, and aging-induced performance deterioration of SBS-modified asphalt under high–low temperature cycling and long-term thermo-oxidative aging [6,7,8]. In terms of macroscopic performance assessment, Wang et al. [9] proposed innovative field-testing methods and clarified the key performance requirements for waterproofing materials subjected to substrate deformation. Garrido et al. [10] quantified the effects of temperature conditioning on tensile strength and joint peel strength. Dong et al. [11] conducted freeze–thaw cycling tests on SBS-modified asphalt waterproofing membranes with different asphalt mixture proportions and revealed their degradation characteristics under low-temperature service conditions, thereby providing guidance for material selection and construction in cold regions. Ameri et al. [12] further investigated the deterioration of these materials under coupled thermo-oxidative aging and freeze–thaw cycling, demonstrating that oxidation and microcrack propagation are major contributors to performance degradation. Qin et al. [13] examined the temperature-dependent peel performance and thermal stability of membrane joints and proposed recommendations for the application of different modified asphalt systems under various temperature conditions. Liu et al. [14] further demonstrated that aging significantly alters the rheological behavior of asphalt binders and weakens asphalt–filler interactions, while Song et al. [15] revealed that natural aging in cold and arid environments induces similar chemical evolution to laboratory aging, with ultraviolet radiation playing a dominant role. In addition, Zhou et al. [16] and Zhang et al. [17] highlighted that membrane manufacturing parameters and interfacial compatibility critically affect the thermal aging resistance, low-temperature flexibility, and long-term repair performance of waterproofing systems. In addition, regarding material compatibility in engineering repair, Wu [18] and Zhang et al. [19] elucidated the interfacial synergistic effects and fatigue degradation behavior, providing important support for the durability design and long-term service performance of waterproofing systems.

Despite these fruitful achievements, a critical theoretical gap persists: current discussions on low-temperature flexibility predominantly rely on the “steady-state thermal equilibrium” assumption, with a dearth of in-depth analyses concerning the transient heat conduction kinetics of multi-layer composites in extreme cold-shock environments [20,21]. During the initial stage of standardized cold shock (−25 °C liquid phase intrusion), pronounced transient temperature gradients and heat transfer lag phenomena manifest within the membrane due to the significant discrepancies in thermophysical properties between the asphalt coating and the reinforcement matrix. This non-homogeneous spatial evolution of the internal temperature field dictates the concentration of localized thermal stress and the initiation of microscopic mechanical damage. However, the lack of cross-validation based on high-frequency in situ measurements and three-dimensional numerical simulations leads to an unquantifiable deviation between current thermodynamic boundary conditions and the actual thermodynamic state within the material.

Addressing these gaps, this paper endeavors to reveal the heat conduction mechanisms of waterproofing membranes under transient cold shocks and their subsequent influence on low-temperature flexibility evolution. Focusing on commonly utilized 3 mm and 4 mm SBS-modified and root-resistant asphalt membranes, micro-temperature sensors were accurately pre-embedded in situ to measure dynamic temperature responses in a −25 °C environment. Simultaneously, a three-dimensional transient heat transfer numerical model was constructed using the Finite Element Method (FEM). Through multi-dimensional cross-validation, the heat transfer lag time at the critical internal interface and the evolution laws of spatial temperature gradients were precisely quantified. This research delineates the causal chain of “transient heat transfer dynamics–material service response,” establishing precise thermodynamic boundaries for analyzing low-temperature embrittlement and providing a theoretical foundation for the durability design and service state evaluation of waterproofing infrastructure in cold regions.

## 2. Experimental

### 2.1. Materials

To systematically investigate the influence of interfacial heterogeneity—specifically coating thickness, matrix composition, and composite layering—on internal transient heat conduction kinetics, three representative categories of waterproofing membranes were selected as test subjects. These comprised conventional elastomeric (SBS) modified bituminous membranes with thicknesses of 3 mm and 4 mm, and a root-resistant modified bituminous membrane with a thickness of 4 mm. All raw materials used for preparing the waterproofing membrane specimens were supplied by Oriental Yuhong Waterproof Technology Co., Ltd., Beijing, China. To ensure statistical validity and reproducibility, five replicate specimens were prepared for each specification. The detailed physical properties and compositional characteristics for all specimens are summarized in Table 1.

It should be noted that the results of this study are mainly applicable to the selected SBS-modified bitumen membranes and root-resistant modified bitumen membranes. Although five samples were tested for each material to ensure repeatability, membranes produced with other bitumen grades, modifier systems, or reinforcement structures may exhibit different cooling rates, thermal lag, and microstructural responses. Therefore, further verification is required before directly extending the quantitative results to other types of bituminous waterproofing membranes.

### 2.2. Specimen Preparation and Sensor Embedding

#### 2.2.1. Specimen Preparation

**Figure 1:**
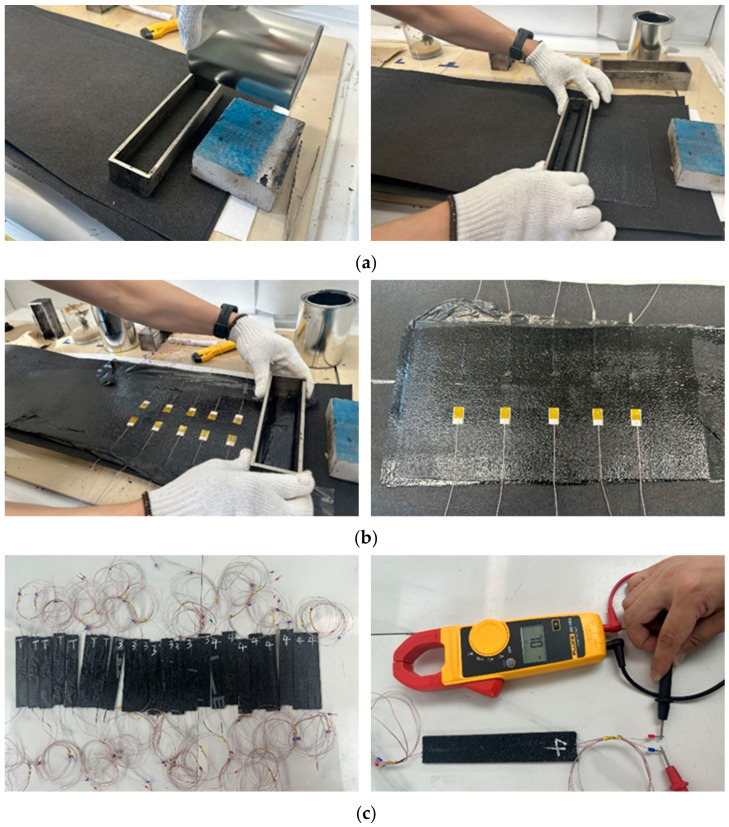
Preparation process of membrane specimens. (**a**) Lower-substrate casting. (**b**) Topological sensor integration. (**c**) Encapsulation and structural validation.

Standard specimens integrated with micro-temperature sensors were prepared using a precision layered-scraping and in situ encapsulation procedure to reproduce the internal structure of composite waterproofing membranes and enable transient temperature monitoring under cryogenic conditions. The equipment used for specimen preparation included a thermostatic heating platform, a customized stainless-steel scraping/extrusion mold, a digital thickness gauge, a precision cutting device, positioning fixtures, and a high-precision digital multimeter. Factory-standardized SBS-modified bitumen was melted and homogenized on the thermostatic heating platform, while polyester or copper substrates were used as reinforcement frameworks. The customized scraping/extrusion mold, equipped with a calibrated notch height, was used to control the coating thickness during layered casting. The final thickness of each specimen was verified with the digital thickness gauge, and the specimens were cut to the required dimensions using the precision cutting device. During sensor embedding, tweezers and positioning fixtures were used to maintain the designed sensor layout. After encapsulation, the continuity of each embedded sensor was verified using the digital multimeter to confirm that the sensors remained intact during fabrication. Finally, the specimens were sealed with a polyethylene isolation film before low-temperature testing. The detailed fabrication process is illustrated in Figure 1.

(a) Lower-substrate casting: A customized extrusion mold with a calibrated notch height was employed to uniformly apply the hot-melt SBS-modified bitumen onto the surface of the pre-conditioned, horizontally aligned substrate.
(b) Topological sensor integration: Once the lower bitumen reached a visco-plastic state, the sensor network was deployed. For 3 mm thick specimens, a coplanar horizontal configuration was adopted for dual-point sensor implantation. For 4 mm thick specimens and those featuring copper substrates, a staggered multi-layer embedding technique was utilized to mitigate localized thermal-resistance perturbations and spatial constraints. Specifically, following the encapsulation of the primary sensor row with 0.5 mm of bitumen, the secondary row was positioned at a longitudinal interval of ≥30 mm.
(c) Encapsulation and structural validation: The bituminous coating on the reverse side was leveled to the nominal thickness (1.0 mm or 1.5 mm) and immediately sealed with a polyethylene (PE) isolation film to eliminate interfacial voids. Following precision cutting, a continuity test was performed using a high-precision digital multimeter to verify the electromechanical integrity of all embedded micro-sensors throughout the fabrication process.

#### 2.2.2. Sensor Embedding

Given the significant disparities in thermophysical properties—specifically thermal conductivity and specific heat capacity—between the asphalt coating and the reinforcement base within composite waterproofing membranes, the transient heat transfer process at the interface exhibits pronounced thermal hysteresis. The non-uniform thermal contraction and localized stress concentration triggered by steep temperature gradients at this interface are often the governing factors driving interfacial debonding and overall brittle fracture of the material.

**Figure 2:**
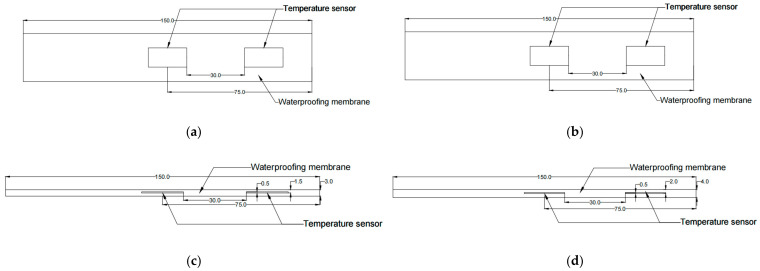
Temperature sensor arrangement. (**a**) Front view of the 3 mm specimen. (**b**) Front view of the 4 mm specimen. (**c**) Side view of the 3 mm specimen. (**d**) Side view of the 4 mm specimen.

Consequently, this study departs from conventional macroscopic surface measurement techniques and innovatively adopts the principle of “critical thermal conduction point control.” Micro-temperature sensors were precisely pre-embedded in situ at the interface between the asphalt coating and the reinforcement base; the detailed spatial configuration of these sensors is illustrated in Figure 2.

**Figure 3:**
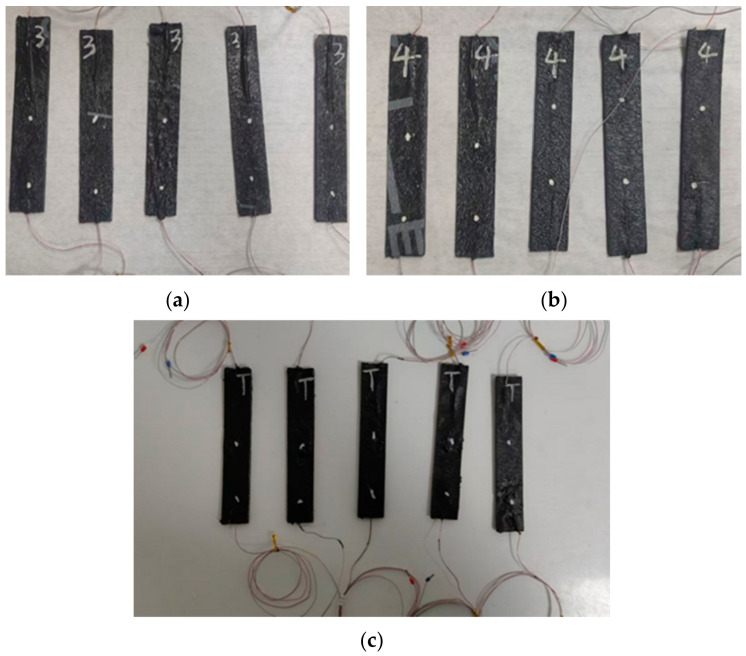
Spatial layout and configuration of the test specimens. (**a**) The 3 mm modified bitumen waterproofing membrane specimen. (**b**) The 4 mm modified bitumen waterproofing membrane specimen. (**c**) The 4 mm elastomeric SBS-modified bitumen root-resistant waterproofing membrane specimen.

The specimens were prepared according to the above method and with the sensor positions arranged as shown in Figure 3. To ensure the accuracy and reproducibility of the sensor arrangement, the geometric dimensions of the specimens and the sensor locations were strictly controlled during specimen preparation. The specimens were cut according to the designed dimensions, with the length deviation controlled within ±1 mm and the thickness deviation controlled within ±0.1 mm. Before encapsulation, the final thickness was verified at multiple representative locations using a digital thickness gauge. The micro-temperature sensors were positioned at the designed bitumen–reinforcement interface using positioning fixtures, and the deviation of the sensor location was controlled within ±0.1 mm.

### 2.3. Experimental Setup and Testing Procedure

#### 2.3.1. Experimental Design

The experiment was designed to evaluate the transient internal temperature response of composite bituminous waterproofing membranes under a uniform low-temperature boundary condition. Based on the specimen configurations described in Section 2.1 and Section 2.2, three groups of membranes were tested to distinguish the effects of membrane thickness and material type on heat transfer lag. All specimens were subjected to the same liquid-phase cooling condition at −25 °C, and the internal temperature was continuously recorded for 60 min. The temperature–time histories obtained from the embedded sensors were used to determine the cooling characteristics, thermal lag behavior, and time required for the specimens to approach thermal equilibrium. The experimental arrangement is summarized in Table 2.

#### 2.3.2. Experimental Setup

**Figure 4:**
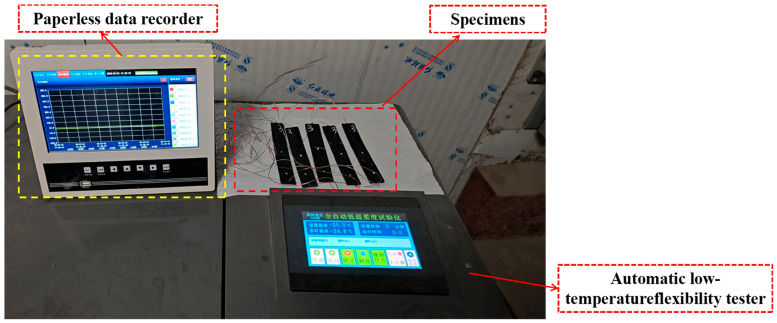
Experimental apparatus.

To accurately simulate and quantify the evolution of the transient temperature field within composite waterproofing membranes under low-temperature conditions, a dynamic temperature monitoring system was established, as illustrated in Figure 4. The experimental system mainly consisted of a fully automatic low-temperature flexibility tester, a liquid-phase cooling medium, embedded micro-temperature sensors, and a paperless data recording unit. The fully automatic low-temperature flexibility tester was manufactured by Tianjin Meters Testing Machine Factory, Tianjin, China, and was used to provide a stable cryogenic environment for low-temperature conditioning and flexibility testing. The temperature control range of the tester was −5 to −45 °C. An aqueous glycerol solution was used as the liquid-phase cooling medium to ensure stable contact heat transfer under sub-zero conditions.

The transient temperature response inside the specimens was monitored using embedded micro-temperature sensors with an accuracy of ±0.1 °C. Temperature signals were continuously recorded using a paperless data recorder at a recording frequency of 1–10 Hz. During testing, the specimens were fully immersed in the cooling medium to ensure uniform liquid–solid contact, and the sensor leads were sealed to prevent liquid penetration and signal disturbance.

#### 2.3.3. Testing Procedure

The experimental procedure adhered to current testing standards for the low-temperature flexibility of waterproofing materials. The target boundary temperature of the cooling medium was equilibrated at −25 °C. The laboratory ambient temperature and relative humidity were maintained at 23°C and 50%, respectively. During the loading phase, hermetically sealed specimens with integrated sensors were rapidly immersed in the cryogenic bath to simulate idealized transient low-temperature conditions. Starting from the initial contact (t = 0) with the liquid-phase boundary, the multi-channel high-frequency DAQ system was synchronously triggered for continuous in situ tracking over a duration of 60 ± 5 min. This full-time-domain monitoring strategy was designed to quantitatively evaluate the thermal lag at the critical internal point as it converged toward the target ambient temperature, the thermodynamic stability during the isothermal phase, and the spatio-temporal evolution of internal temperature gradients.

**Figure 5:**
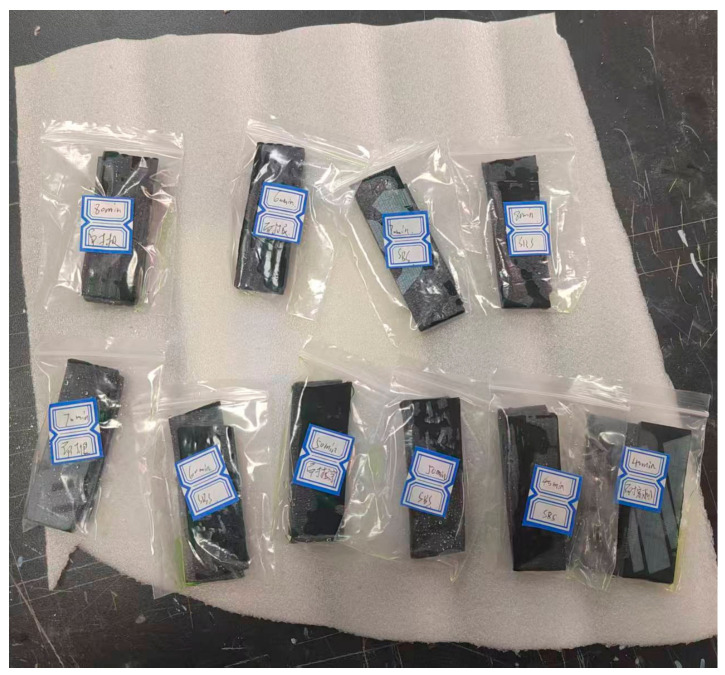
Specimens under different cooling durations.

Furthermore, to complement the macroscopic thermal analysis, scanning electron microscopy (SEM) was performed to examine the micro-morphological evolution of the membranes after low-temperature conditioning. Representative specimens were collected after cooling for 40, 50, 60, 70, and 80 min, as shown in Figure 5. The specimens were cryo-fractured to expose the cross-sectional interface between the bituminous matrix and the reinforcement layer, and the fractured surfaces were sputter-coated with gold to improve electrical conductivity. SEM observations were conducted using a scanning electron microscope (Model FE-STEM SU9000, Hitachi, Tokyo, Japan) at an accelerating voltage of 1 kV. The interfacial morphology between the bituminous matrix and the reinforcement layer was observed at selected magnifications to identify microcrack initiation, crack propagation, and interfacial debonding induced by low-temperature thermal shock. All SEM micrographs were acquired at the same magnification of 220×. These micrographs provided microscopic evidence for interpreting the mesoscopic embrittlement behavior and interfacial degradation mechanisms of bituminous membranes under transient thermal stress.

### 2.4. Numerical Modeling

A three-dimensional transient heat transfer model was established to simulate the internal temperature-field evolution of the composite bituminous waterproofing membranes during low-temperature immersion. The numerical model was developed using MIDAS FEA NX software (version 2023), and the transient heat transfer module was adopted for the simulation. The model geometry was constructed according to the nominal dimensions and layered configuration of the tested specimens. The bituminous coating layer and reinforcement layer were explicitly represented to account for the heterogeneous thermal properties of the composite membrane.

#### 2.4.1. Governing Equation and Assumptions

The transient heat conduction process was governed by Fourier’s law and the energy conservation equation. In the absence of internal heat generation, the governing equation can be expressed as

$$\rho c_{p}\frac{\partial T}{\partial t}=\frac{\partial }{\partial x}\left(\lambda_{x}\frac{\partial T}{\partial x}\right)+\frac{\partial }{\partial y}\left(\lambda_{y}\frac{\partial T}{\partial y}\right)+\frac{\partial }{\partial z}\left(\lambda_{z}\frac{\partial T}{\partial z}\right)$$

In the equation, ρ represents the material density $\left(kg/m^{3}\right)$; $c_{p}$ is the specific heat capacity (J/(kg·K); T is the temperature variable (°C); t denotes the conduction time; and $\lambda_{x},\;\lambda_{y},\lambda_{z}$ are the thermal conductivities (W/(m·K)) in the three orthogonal directions, respectively. Under the assumption of isotropy, ($k_{x}=k_{y}=k_{z}=k$).

#### 2.4.2. Initial and Boundary Conditions

The initial temperature of the entire computational domain was set according to the measured initial specimen temperature before immersion:

$$T(x,y,z,0)=T_{0}.$$

where $T_{0}$ is approximately 28.0–28.5 °C. During immersion, all external surfaces of the specimen were subjected to convective heat transfer with the liquid-phase cooling medium. The boundary condition was defined as

$$-k\nabla T\cdot n=h(T_{s}-T_{\infty }).$$

where n is the outward normal vector, h is the convective heat transfer coefficient, $T_{s}$ is the specimen surface temperature, and $T_{\infty }$ is the cooling-medium temperature. The cooling-medium temperature was set to −25 °C, and the convective heat transfer coefficient was set to 200 W/(m^2^·K) to represent the liquid–solid heat exchange during full immersion.

#### 2.4.3. Material Properties and Interface Contact

The bituminous coating, polyester reinforcement, and copper substrate were modeled as isotropic heat-conducting materials with different thermophysical properties. The density, specific heat capacity, and thermal conductivity used in the model are listed in Table 3. Since the specimens were prepared by layered casting and in situ encapsulation, the interfaces between the bituminous coating and the reinforcement layer were assumed to be fully bonded. Perfect thermal contact was therefore imposed at the mating surfaces, and no additional interfacial thermal resistance was considered. The thermal parameters used in the model were selected based on the material composition and available reference values, and the equivalent thermal conductivity was used to represent the overall heat transfer behavior of the multi-layer membrane system.

## 3. Results and Discussion

### 3.1. Temperature-Field Analysis of Membrane Low-Temperature Flexibility

**Figure 6:**
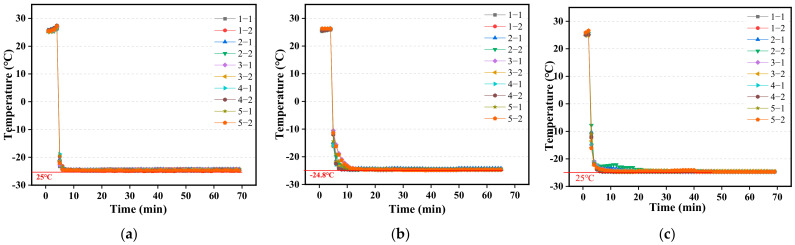
Temperature evolution. (**a**) The 3 mm modified bitumen waterproofing membrane. (**b**) The 4 mm elastomeric SBS-modified bitumen root-resistant waterproofing membrane. (**c**) The 4 mm root-resistant membrane.

Figure 6 illustrates the transient temperature evolution for three different specifications of waterproofing membranes subjected to an ambient temperature of −25 °C. The experimental data indicate that prior to testing, the initial isothermal state of all specimens was precisely maintained within the range of 28.0 to 28.5 °C, ensuring rigorous consistency in the initial thermodynamic boundary conditions. During the initial cooling phase (0 to 10 min) upon immersion, a substantial thermal gradient of approximately 53 °C triggered intense convective heat transfer between the material surface and the liquid medium. Driven by this steep boundary gradient, the temperature monitored at the bitumen–reinforcement interface exhibited a characteristic nonlinear decay. As internal thermal energy dissipated to the external environment, the diminishing temperature gradient resulted in pronounced thermal hysteresis and a gradual attenuation of the interfacial cooling rate. Following 20 min of transient heat transfer, the temperature–time curves for all membrane specifications asymptotically converged toward a quasi-steady thermodynamic equilibrium near −24.8 °C, closely approaching the target coolant temperature.

This process confirms that the 60 min cooling cycle prescribed by current testing standards provides an adequate thermodynamic equilibrium window to eliminate internal temperature gradients caused by structural heterogeneity (e.g., multiple interfaces and varying thermal resistances). This ensures that composite membranes, regardless of thickness or reinforcement type, achieve a uniform and stable isothermal state before proceeding to macroscopic mechanical characterization.

The temperature monitoring results in Figure 6 were obtained from five repeated tests for each membrane sample. The repeated curves showed a high degree of overlap throughout the cooling process, indicating good repeatability of the experimental temperature measurements. Therefore, the observed cooling trend and temperature-equilibrium behavior were considered reliable for subsequent comparison with the numerical simulation results.

### 3.2. Finite Element Simulation Analysis of Waterproofing Membrane Specimens

#### 3.2.1. Model Construction

**Figure 7:**
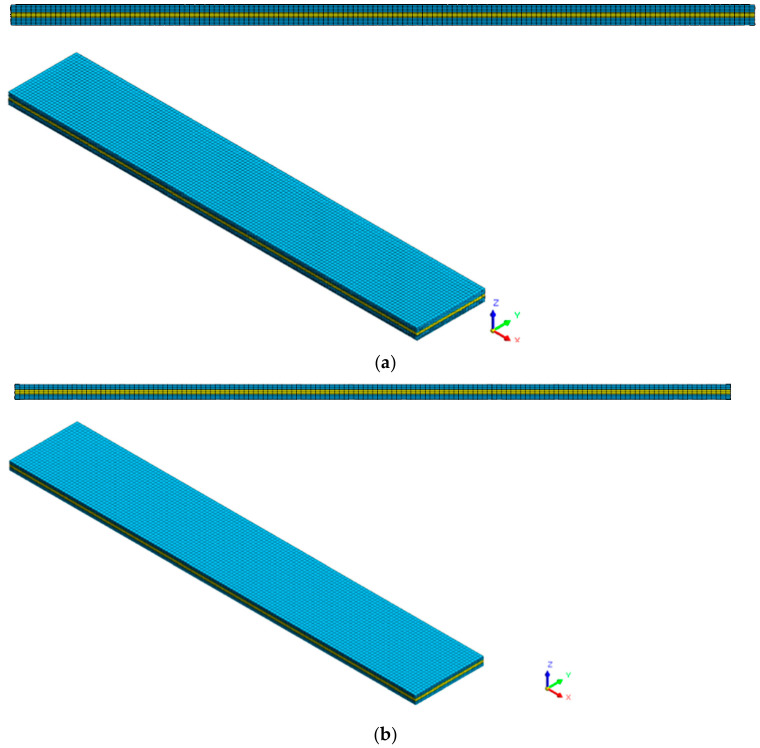
3D finite element temperature-field models of SBS-modified bitumen membranes. (**a**) The 4 mm elastomeric SBS-modified bitumen membrane. (**b**) The 3 mm elastomeric SBS-modified bitumen membrane.

The initial global temperature field of the model was prescribed as 28 °C. Convective boundary conditions (specifically, Robin boundary conditions) were applied to the external surfaces to simulate the instantaneous immersion into a cryogenic environment, where the ambient temperature drops abruptly to −25 °C under intense convective heat exchange. The resulting Midas finite element (FE) model and its discretized mesh configuration are illustrated in Figure 7.

#### 3.2.2. Simulation Results and Analysis

**Figure 8:**
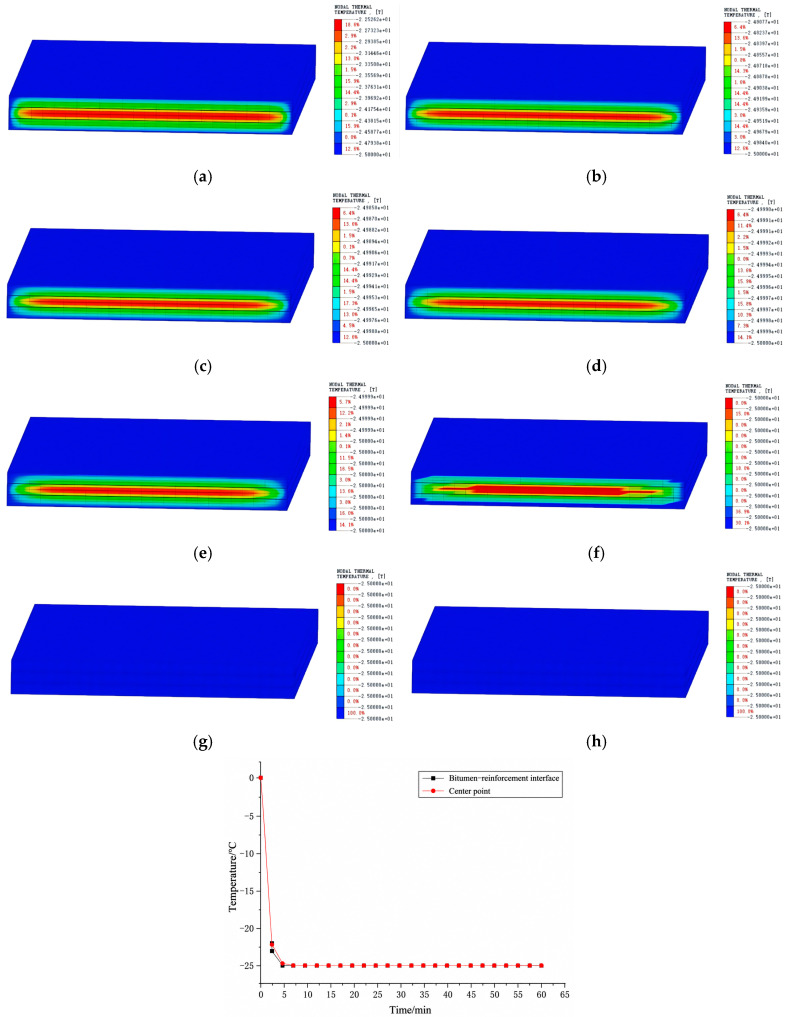
The spatio-temporal evolution of the temperature for the 4 mm elastomeric SBS-modified bitumen membrane. (**a**) Cross-sectional temperature distribution at 2 min. (**b**) Cross-sectional temperature distribution at 4 min. (**c**) Cross-sectional temperature distribution at 6 min. (**d**) Cross-sectional temperature distribution at 8 min. (**e**) Cross-sectional temperature distribution at 10 min. (**f**) Cross-sectional temperature distribution at 12 min. (**g**) Cross-sectional temperature distribution at 14 min. (**h**) Cross-sectional temperature distribution at 16 min.

(1) The 4 mm elastomeric SBS-modified bitumen membrane:

The spatio-temporal evolution of the cross-sectional temperature contour plots was extracted for the interval between 2 min and 16 min. Additionally, the transient cooling curves at critical interfacial nodes were derived to quantify the thermal response over time; these results are illustrated in Figure 8.

**Figure 9:**
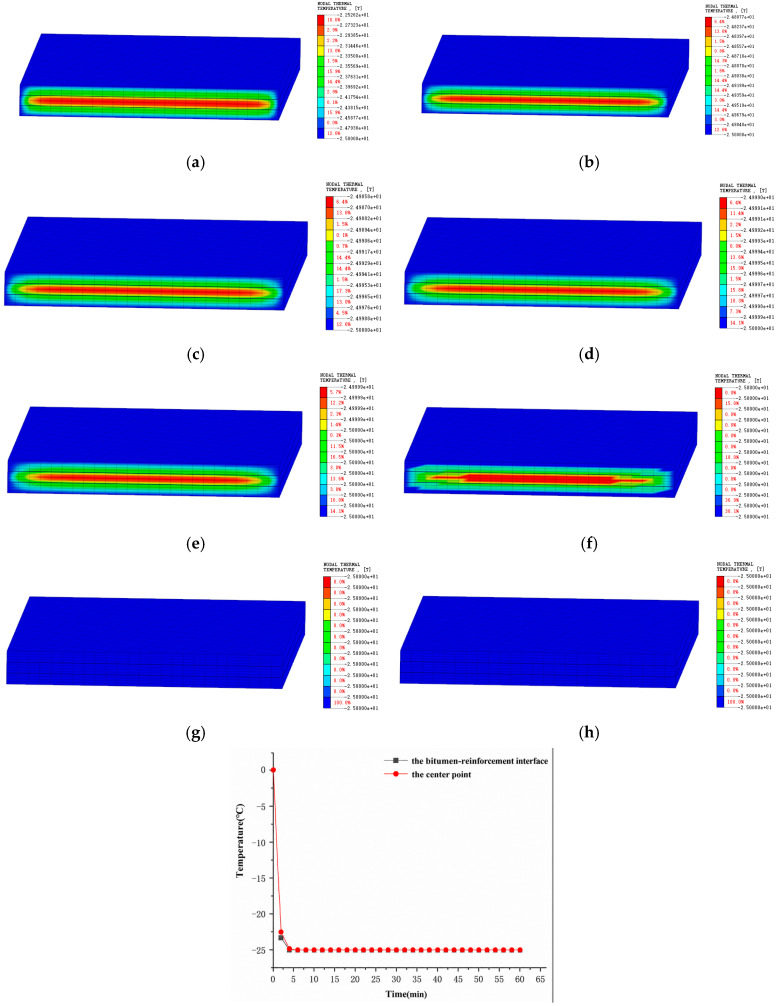
The spatio-temporal evolution of the temperature for the 3 mm elastomeric SBS-modified bitumen membrane. (**a**) Cross-sectional temperature distribution at 2 min. (**b**) Cross-sectional temperature distribution at 4 min. (**c**) Cross-sectional temperature distribution at 6 min. (**d**) Cross-sectional temperature distribution at 8 min. (**e**) Cross-sectional temperature distribution at 10 min. (**f**) Cross-sectional temperature distribution at 12 min. (**g**) Cross-sectional temperature distribution at 14 min. (**h**) Cross-sectional temperature distribution at 16 min.

(2) The 3 mm elastomeric SBS-modified bitumen membrane:

The spatio-temporal evolution of the cross-sectional temperature contour plots was extracted for the interval between 2 min and 16 min. Additionally, the transient cooling curves at critical interfacial nodes were derived to quantify the thermal response over time; these results are illustrated in Figure 9.

**Figure 10:**
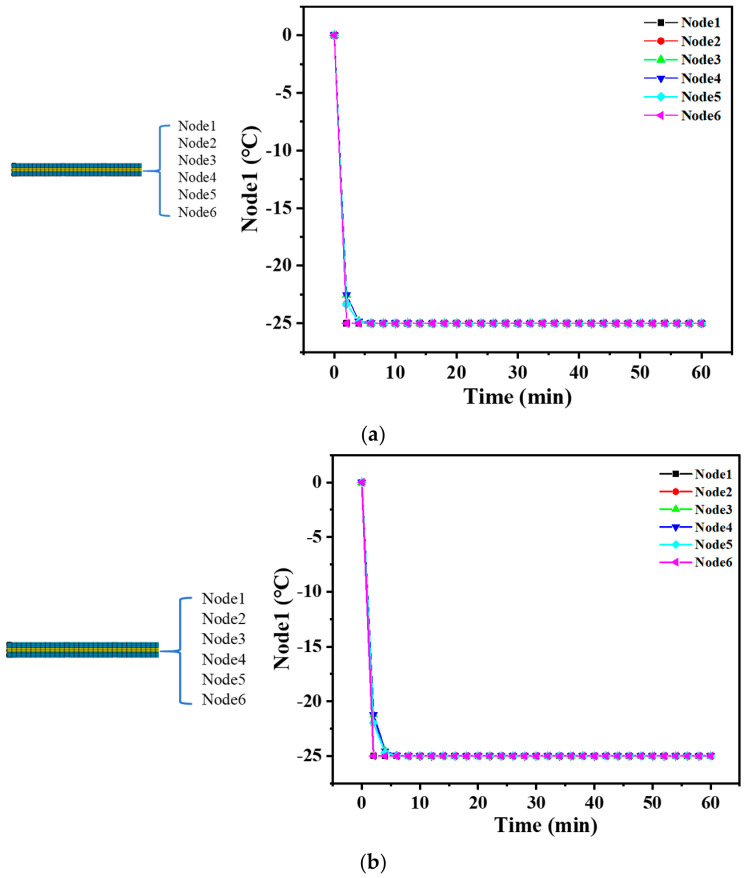
Temperature profiles across the membrane thickness at different exposure times. (**a**) The 3 mm elastomeric SBS-modified bitumen membrane. (**b**) The 4 mm elastomeric SBS-modified bitumen membrane.

To provide a more quantitative interpretation of the temperature-field evolution, temperature profiles along the thickness direction were extracted from the central cross-section of the numerical model at selected exposure times, as is shown in Figure 10. Compared with the temperature isofields, these sectional profiles clearly demonstrate the surface-to-core cooling sequence and the progressive attenuation of the through-thickness temperature gradient. During the initial 2–6 min, the surface temperature decreased rapidly because of direct contact with the liquid cooling medium, whereas the interfacial and core regions remained at significantly higher temperatures. The maximum through-thickness temperature difference occurred during the early cooling stage, indicating the strongest transient thermal non-uniformity. With increasing exposure time, the temperature profiles gradually flattened, suggesting the dissipation of the internal thermal gradient and the approach toward thermal equilibrium.

#### 3.2.3. Analysis of Finite Element Model Results

Based on the established three-dimensional transient finite element model, cross-sectional temperature contours were extracted from 2 to 16 min to illustrate the characteristic surface-to-core evolution of heat transfer within the composite membrane. During the initial cooling stage (0–5 min), the near-surface and peripheral regions cooled rapidly because of strong convective heat exchange at the liquid boundary. In contrast, heat dissipation in the interior was delayed owing to the low thermal diffusivity of the polymer-modified bitumen and the reinforcement layer, resulting in a pronounced thermal lag at the coating–reinforcement interface.

This interfacial thermal lag led to substantial transient temperature gradients across the membrane thickness, particularly within the first 10 min of cooling. From a micromechanical perspective, such non-uniform temperature evolution may induce incompatible thermal contraction between adjacent layers and thereby generate localized thermal stresses at the interface. These stresses can act as precursors to interfacial debonding, microcrack initiation, and eventual brittle fracture under low-temperature conditions.

To validate the numerical model, the temperature–time histories at critical interfacial nodes were compared with the in situ measurements obtained from the embedded micro-temperature sensors. The simulated results showed good qualitative agreement with the experimental cooling curves, reproducing the main features of the transient thermal response, including the rapid initial temperature drop, the subsequent reduction in cooling rate, and the gradual approach to thermal equilibrium. Although minor deviations were present because of the idealized assumptions adopted in the model, such as material homogeneity and temperature-independent properties, the comparison confirms that the FE model can reasonably capture the transient internal heat transfer behavior and interfacial thermal lag of the composite membranes. These results also support the adequacy of the 60 min conditioning period specified in the current testing standard for minimizing residual internal temperature gradients prior to mechanical characterization.

A comparative analysis of the different membrane samples was further conducted based on the simulated and measured temperature histories. The results indicate that the tested samples exhibited broadly similar cooling trends under the same −25 °C low-temperature conditioning condition. All samples showed a rapid temperature decrease during the initial cooling stage, followed by a gradual reduction in cooling rate and final approach to thermal equilibrium. Only minor differences were observed among the samples, mainly reflected in slight variations in cooling rate and interfacial temperature lag. This suggests that, within the investigated conditioning period, the effects of sample specification, thickness, and material structure on the overall temperature-field evolution were present but not substantial.

### 3.3. Micro-Morphological Validation and Defect Initiation Analysis

It should be noted that defect initiation was not identified solely from the temperature contours. Instead, the temperature field was used to determine the high-risk regions and exposure periods for defect initiation. In the present study, potential defect initiation was associated with two thermal conditions: (i) the presence of a large transient through-thickness temperature gradient, which may induce incompatible thermal contraction between the bituminous coating and the reinforcement layer; and (ii) the decrease in the interfacial temperature to the low-temperature embrittlement range, where the asphalt–polymer matrix loses deformation compatibility. The actual occurrence of defects was then verified by SEM observations, where microcrack initiation, interfacial debonding, exposed reinforcement, and fiber or additive pull-out were used as microscopic evidence of damage.

**Figure 11:**
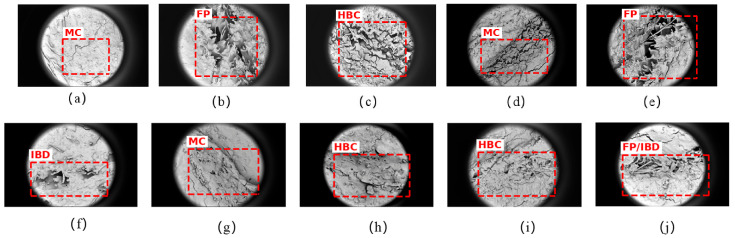
Annotated SEM images of typical defect zones in bituminous membranes after different cooling durations at the same magnification of 220×. Red dashed boxes indicate highlighted defect regions; MC, IBD, HBC, and FP denote microcrack, interfacial debonding, heterogeneous brittle cleavage, and fiber/additive pull-out, respectively. For root-resistant modified bitumen membranes, (**a**) 40 min, MC; (**b**) 50 min, FP; (**c**) 60 min, HBC; (**d**) 70 min, MC; and (**e**) 80 min, FP. For SBS-modified bitumen membranes, (**f**) 40 min, IBD; (**g**) 50 min, MC; (**h**) 60 min, HBC; (**i**) 70 min, HBC; and (**j**) 80 min, FP/IBD.

To further validate the relationship between transient thermal response and microstructural evolution, SEM was employed to characterize the cryo-fractured cross-sections of the membranes after different cooling durations. Figure 11 shows the SEM images of the 4 mm root-resistant modified bitumen membrane and the SBS-modified bitumen membrane after cooling for 40, 50, 60, 70, and 80 min. The main defect zones, including microcracks, interfacial debonding, heterogeneous brittle cleavage, and fiber/additive pull-out, are marked by red dashed boxes and abbreviated labels in the SEM images. Linear and sharp discontinuities in the asphalt matrix were identified as microcracks. Gaps or separations along the bitumen–reinforcement interface were identified as interfacial debonding. Rough and irregular fracture ridges were classified as heterogeneous brittle cleavage. Exposed filaments, holes, or elongated cavities left in the matrix were identified as fiber/additive pull-out.

To clarify the relationship between the validated temperature-field evolution and the SEM-observed morphology, the SEM results at 40, 50, and 60 min were compared with the corresponding thermal response characteristics, as summarized in Table 4.

For the root-resistant modified bitumen membrane, the fracture surfaces at 40 and 50 min exhibited relatively disordered micro-textures and non-uniform brittle cleavage features. Since the temperature measurements and numerical simulation indicated that the membrane had already approached the low-temperature boundary condition during this period, these heterogeneous morphologies should not be interpreted as evidence of a still-developing macroscopic temperature gradient. Instead, they suggest that the asphalt matrix, polymer phase, and reinforcement interface were undergoing microstructural stabilization under the low-temperature state. The local roughness and irregular cleavage features indicate non-uniform embrittlement and local interfacial mismatch.

At 60 min, the fracture morphology became more homogeneous and compact. The fracture ridges were more continuous, and no obvious large-scale interfacial separation or macro-defect initiation was observed. This morphology is consistent with the temperature-field results, which showed that the internal temperature field had reached a nearly uniform low-temperature state. Therefore, the SEM evidence supports the conclusion that the 60 min conditioning period is sufficient for minimizing the influence of transient heat transfer lag before low-temperature flexibility evaluation.

For the SBS-modified bitumen membrane, the fracture surfaces at 40 and 50 min showed local heterogeneity, including irregular micro-wrinkles, roughened fracture ridges, and non-uniform cleavage features. These features indicate that the SBS-modified asphalt matrix had entered a brittle state, while local phase heterogeneity and interfacial constraint still affected the fracture morphology. After cooling for 60 min, the fracture surface became smoother and denser, suggesting a more uniform frozen state of the asphalt–polymer matrix. This transition agrees with the numerical and experimental temperature results, confirming that the thermal lag effect had been substantially reduced within the standard 60 min conditioning duration.

The specimens cooled for 70 and 80 min showed more severe microstructural deterioration, including exposed reinforcement, fiber/additive pull-out, and local loss of matrix continuity. These extended-duration observations were not used for direct validation of the numerical temperature-field model because the numerical–experimental comparison in this study focused on the standard 60 min conditioning period. Instead, the 70 and 80 min SEM results indicate that prolonged cryogenic exposure after thermal equilibrium may further weaken the bonding capacity of the asphalt matrix and promote accumulated brittle damage.

### 3.4. Comparison with Previous Studies and Limitations

Previous studies have mainly focused on the low-temperature mechanical performance, freeze–thaw deterioration, aging behavior, and microstructural degradation of SBS-modified bitumen and waterproofing membranes. These studies have shown that low temperature and environmental aging can promote brittle fracture, microcrack formation, phase deterioration, and interfacial weakening. Compared with these studies, the present work focuses on the transient internal heat transfer process during low-temperature conditioning. The in situ temperature measurements and finite element simulation reveal a surface-to-core cooling sequence and interfacial thermal lag, which provide a thermal explanation for the importance of sufficient low-temperature conditioning time before flexibility evaluation.

The SEM observations in this study are generally consistent with previously reported microstructural degradation mechanisms, such as brittle cleavage, local cracking, interfacial mismatch, and loss of matrix bonding capacity. However, this study further links these microstructural features with the measured and simulated temperature-field evolution, especially within the standard 60 min conditioning period.

Several limitations should be noted. First, the experiments were conducted at a single cooling temperature of −25 °C, and the effects of other temperatures or cyclic cooling conditions were not considered. Second, the numerical model was limited to transient heat transfer and did not include coupled thermo-mechanical stress or damage evolution. Third, perfect thermal contact and temperature-independent material properties were assumed in the model. Finally, the SEM analysis was mainly qualitative. Future work should include additional temperature levels, quantitative SEM image analysis, and coupled thermal–mechanical simulations.

## 4. Conclusions

This study presents a systematic investigation into the internal heat transfer mechanisms and mesoscopic degradation evolution of multi-layer composite bitumen waterproofing membranes under low-temperature conditions. Through the cross-validation of in situ micro-sensor measurements, 3D non-steady-state FEM simulations, and multi-scale SEM characterization, the spatio-temporal evolution of dynamic internal temperature fields and their corresponding micro-morphological responses were precisely quantified. The primary conclusions are as follows:

(1) A pronounced surface-to-interior transient thermal lag effect was identified in composite membranes subjected to −25 °C cooling. Due to the low thermal diffusivity of the bitumen coating and reinforcement layers, surface convective heat transfer peaks within the initial 10 min; however, the internal core interfaces—acting as high-thermal-resistance zones—require 10–20 min of nonlinear heat dissipation to progressively converge toward the target ambient temperature.
(2) Prior to reaching global thermodynamic equilibrium during the initial cooling phase, intense spatial temperature gradients inevitably develop within the membrane. These gradients drive incompatible thermal contraction across the composite layers. External loading applied during this unsteady state is highly likely to trigger unanticipated microcrack initiation and premature brittle fracture due to the acute concentration of localized thermal stresses at the interfaces.
(3) Sequential SEM fracture analysis indicates that the 60 min low-temperature immersion period corresponds to the formation of a relatively stable frozen morphology of the asphalt–polymer matrix and the substantial reduction in transient thermal lag. The SEM observations at 40–60 min are consistent with the experimental and numerical temperature-field results, whereas the additional observations at 70 and 80 min reveal accumulated microstructural degradation under prolonged cryogenic exposure after thermal equilibrium. These findings support the rationality of the 60 min isothermal conditioning period stipulated in current testing standards and provide a basis for reducing mechanical testing deviations caused by non-uniform cooling.

Future research should focus on different cooling temperatures, cyclic low-temperature conditions, and membranes with other bitumen grades, modifier systems, and reinforcement structures. Coupled thermal–mechanical modeling and quantitative SEM image analysis should also be conducted to further clarify the relationship among temperature-field evolution, thermal stress, and microstructural damage.

## Figures and Tables

**Table 1:** Specifications of specimens for low-temperature flexibility temperature-field testing.

No.	Material Type	Thickness (mm)	Dimensions	Reinforcement Matrix	Surface Material	Quantity
1	SBS-modified bitumen	3	150 mm × 25 mm	Polyester felt	PE film/sand/mineral granules	5
2	SBS-modified bitumen	4		Polyester felt	PE film/sand/mineral granules	5
3	Root-resistant bitumen	4		Copper/polyester composite matrix	PE film/sand/mineral granules	5

**Table 2:** Summary of the experimental arrangement.

Group	Specimen	Thickness	Number	Cooling Temperature	Recording Duration
G1	SBS-modified bituminous membrane	3 mm	5	−25 °C	60 min
G2	SBS-modified bituminous membrane	4 mm	5	−25 °C	60 min
G3	Root-resistant modified bituminous membrane	4 mm	5	−25 °C	60 min

**Table 3:** Thermophysical parameters of the materials.

Material	Density ρ (kg/m^3^)	Specific Heat Capacity c_p_ (J/(kg·K))	Thermal Conductivity λ (W/(m·K))
SBS-modified bituminous coating	1000–1050	1800–2100	0.15–0.25
Polyester matrix	1300–1380	1200–1400	0.10–0.18
Copper matrix	8900–8960	380–390	380–400

**Table 4:** Correlation between temperature response and SEM morphology.

Exposure Time	Temperature-Field Characteristic	SEM Observation	Interpretation
40 min	The interface temperature approached the cooling-medium temperature, and the cooling rate became very small.	Heterogeneous brittle cleavage, local roughness, irregular wrinkles, and non-uniform fracture ridges were observed.	The membrane had entered a low-temperature embrittlement state, but local microstructural stabilization was still incomplete.
50 min	The internal temperature field was close to quasi-equilibrium, with only minor local thermal mismatch.	Cleavage features became more continuous, while local heterogeneity remained visible.	The asphalt–polymer matrix was approaching a stable frozen state, but phase/interface heterogeneity still affected the fracture morphology.
60 min	The experimental and numerical results indicated a nearly uniform low-temperature state.	The fracture surface became smoother, denser, and more homogeneous.	The heat transfer lag effect had been substantially reduced, supporting the adequacy of the 60 min conditioning period.

## Data Availability

Data are contained within the article.
